# Psychometric properties of the translated Spanish version of the Pain Self-Efficacy Questionnaire

**DOI:** 10.3389/fmed.2023.1226037

**Published:** 2023-07-03

**Authors:** Borja Perez-Dominguez, Sara Perpiña-Martinez, Isabel Escobio-Prieto, Marta de la Fuente-Costa, Alvaro Manuel Rodriguez-Rodriguez, Maria Blanco-Diaz

**Affiliations:** ^1^Department of Physiotherapy, Exercise Intervention for Health Research Group, University of Valencia, Valencia, Spain; ^2^Faculty of Health Sciences, Pontifical University of Salamanca, Madrid, Spain; ^3^Department of Physiotherapy, Faculty of Nursing, Physiotherapy and Podiatry, University of Seville, Seville, Spain; ^4^Research group CTS-1043: Health, Physiotherapy and Physical Activity, Institute of Biomedicine of Seville (IBIS), Seville, Spain; ^5^Faculty of Medicine and Health Sciences, Physiotherapy and Translational Research Group, Institute of Health Research of the Principality of Asturias, University of Oviedo, Oviedo, Spain

**Keywords:** cross-cultural adaptation, pain self-efficacy questionnaire, reliability, Spain, validity

## Abstract

**Introduction:**

Some patients with rotator cuff injuries do not report significant changes in pain-related outcomes. Pain self-efficacy, which is commonly assessed using the Pain Self-Efficacy Questionnaire, may contribute toward this outcome. However, a Spanish adaptation of this questionnaire is currently lacking. Therefore, this study’s purpose was developing the Spanish version of this questionnaire, and assess its psychometric properties.

**Methods:**

The Spanish version of the Pain Self-Efficacy Questionnaire was translated and culturally adapted, and a sample of 107 patients with rotator cuff injuries completed the questionnaire to examine its convergent validity (analyzing its correlation with the Tampa Scale of Kinesiophobia), its test–retest reliability, for which a subset of 40 participants completed again the questionnaire, and its internal consistency.

**Results:**

Translation was conducted without any problems, and 107 participants completed the study. Mean scores for the Pain Self-Efficacy Questionnaire were 45.2 points (standard deviation, 11.4). The Pain Self-Efficacy Questionnaire showed a moderate negative correlation with the Tampa Scale of Kinesiophobia (Pearson’s correlation index *r* = −0.48) supporting its convergent validity. High test–retest reliability (Intraclass Correlation Coefficient of 0.90) and excellent internal consistency (Cronbach’s *α* value of 0.92) were also found.

**Discussion:**

The Spanish version of the Pain Self-Efficacy Questionnaire presents high validity, test–retest reliability, and internal consistency to assess pain self-efficacy in patients suffering rotator cuff injuries in Spanish-speaking settings.

## 1. Introduction

Rotator cuff injuries account for 80% of primary care consultations for shoulder pain (1), and their incidence increases with age (2). These injuries, among other problems such as weakness and loss of function, can cause significant levels of pain that are crucial when deciding treatment strategies (3). Despite the development of many effective interventions to improve symptoms, around 30% of the patients report no significant change in pain-related outcomes, and psychosocial, occupational and lifestyle factors have been identified as possible reasons for this (1).

One of these factors is pain self-efficacy. Originally defined by Albert Bandura, self-efficacy is one’s confidence or belief in their capacity goal achievement or activity performance (4). Higher levels of self-efficacy are suggested as predictors of better prognosis in patients with musculoskeletal pain (5), less disability, pain, fatigue, or emotional distress (6). Self-efficacy, therefore, determines the willingness to persist when obstacles are faced, avoids shying away from a complicated task, and shows commitment to achieving a goal (7).

The assessment of self-efficacy, as it is a belief, must be self-administered. To assess self-efficacy, many tools have been developed. Some of them are specific to a condition, such as the Arthritis Self-Efficacy Scale (ASES) or the Chronic Disease Self-Efficacy Scale, and some are related to pain and altered pain states, such as the Chronic Pain Self-Efficacy Scale or the Pain Self-Efficacy Scale (PSEQ) (8, 9). When dealing with clinical settings involving musculoskeletal disorders, the PSEQ is the preferred scale used by clinicians (10). It is a short and comprehensive questionnaire composed with 10 items first developed in English, aimed to assess the confidence or ability people with pain have to achieve activities despite their pain (11). The PSEQ assesses several dimensions, including physical functioning, social interaction, and participation in activities of the daily living when pain is present (11).

Translated and adapted versions of the PSEQ have assessed its psychometric properties in several languages, such as Amharic (7), Arabic (12), Canadian-French (1), Catalan (13), Chinese-Hong Kong (14), Chinese-Mainland (15), Danish (16), Italian (17), Farsi (18), Japanese (19), Marathi (20), Mongolian (21), Portuguese-Brazilian (22), Portuguese-European (23), and Yoruba (24). All of these translations were performed rigorously, and several of them adapted items from the questionnaire to their culture. Despite this, a validated version of this questionnaire in Spanish is lacking, limiting its access to healthcare professionals who develop their practice in Spanish-speaking settings. Therefore, this study aimed to translate and culturally adapt the Spanish version of the PSEQ, and examine its psychometric properties.

## 2. Materials and methods

### 2.1. Participants

This cross-sectional study was conducted at a private practice Hospital in Valencia, Spain, and included a sample of patients with rotator cuff injuries that were considered for surgical repair. Recruitment of participants was conducted between March and May 2023. Participants were included based on the following eligibility criteria: (1) adults >18 years old, (2) with a medically diagnosed rotator cuff injury considered for surgical repair, and (3) willingness to participate in the study. Participants were excluded if they had any cognitive impairments that could interfere with the completion of the assessment or if they were illiterate in Spanish. A briefing on the purpose of the study was given to the participants by a member of the research team during consultation with the surgeon, and gave written consent before being considered for enrollment in this study.

### 2.2. Measures

Baseline demographic characteristics from the sample were obtained for descriptive purposes, including sex, age, if the involved side was dominant or not, educational level (uneducated, primary, high school, college) and work status (part-time, full-time, unemployed, or retired).

The PSEQ is a 10-item questionnaire aimed to assess the confidence or ability people with pain have to achieve activities despite their pain. It includes lifestyle, social and daily activity questions the participant has to rate from 0 (not at all confident) to 6 (completely confident) in a Likert-style scale. Scores range from 0 to 60, with higher values indicating stronger self-efficacy levels (11). The psychometric properties, such as a high internal consistency, a high degree of stability, and construct validity of this assessment tool have been previously reported (7, 10).

The Tampa Scale of Kinesiophobia (TSK) was used as a validity criterion. It assesses fear-avoidance behaviors related to pain, and it has been previously translated and adapted in Spanish (25). In each item, patients have to answer in a 4-item Likert-style scale if they strongly agree (1) or strongly disagree (4) with the given statement. The total score ranges from 17 to 68 points, with higher values indicating stronger fear-avoidance behaviors. The preliminary Spanish version of the TSK was reviewed by a group of bilingual experts, including healthcare professionals and researchers familiar with the target population. They assessed the clarity, comprehensibility, and appropriateness of the translated items in the Spanish context. Any necessary modifications or adjustments were made based on their feedback and consensus.

### 2.3. Procedures

#### 2.3.1. Translation and cultural adaptation

Before starting the translation and cross-cultural adaptation process, the original developer of the PSEQ (Professor Michael K. Nicholas) was contacted for the permission to translate and adapt the original English version of the PSEQ into Spanish. Established guidelines must be followed to translate and culturally adapt a questionnaire to a new language and cultural setting (26). The following steps were followed in the translation process: (1) The first author, bilingual in Spanish and English, translated every item from its original English version to Spanish, (2) a forward translation was then conducted twice by two independent authors who were also fluent in both languages, and to solve any differences between those authors, consensus was obtained. Finally (3) a back translation from Spanish to English was conducted twice by two independent authors fluent in both languages. Once the final version of the Spanish translation was approved by every member in the research team through consensus, a preliminary testing for cognitive debriefing was conducted in a small sample of 30 patients with rotator cuff injuries to assess comprehensiveness and clarity of the translated items.

#### 2.3.2. Data collection

Data collection was carried out by two experienced physiotherapist members of the research team from March to May 2023, and was registered in a spreadsheet for further analysis. During their medical visit, participants were asked to complete a short form with their sociodemographic and clinical information, the Spanish version of the PSEQ, and the TSK. To assess test–retest reliability, a subset of 40 participants was asked to complete again the PSEQ within a week after completing it for the first time.

### 2.4. Statistical analysis

To conduct statistical analyses, SPSS Statistics (IBM, Armonk, NY, United States) software, in its 23.0 version for MacOS was used. Baseline demographic information was described as means (standard deviations) for continuous data, and counts (percentages) for categorical data. The Kolmogorov–Smirnov test was used to check for data normal distribution. Missing values were handled using mean imputation. This method involved replacing missing values with the mean value of the available data for each respective variable. Floor and ceiling effects were established and determined by calculating if >15% of the responses given by participants corresponded to the minimum possible score of 0 or the maximum possible score of 60 (27).

#### 2.4.1. Sample size estimation

Published guidelines with established requirements for the validation of a survey-like instrument were followed to conduct the sample size estimation (10 participants per item in the tool) (28). Also, the COSMIN recommendation for the selection of health-status measurement instruments were followed (29), establishing that the sample should at least be seven times the total number of items and ≥ 100. A sample of a minimum of 100 respondents was required, as the PSEQ is a 10-item questionnaire.

#### 2.4.2. Validity

An exploratory factor analysis (EFA) was conducted to assess validity through an exploratory principal component analysis (PCA) with Varimax rotation, establishing the number of generated domains using the Scree test criteria (30). Keyser-Meyer-Olkin (KMO) measure was used for sampling adequacy at >0.8 to be considered good, and the Bartlett test for sphericity was used to determine the level of significance (31, 32). Similar to what previous studies have reported (1, 11–19, 22, 23), a one-factor solution is hypothesized to be found in the factorial analysis.

Additionally, convergent validity was also assessed, and it is defined as how close a measurement tool is related to other measurement tools that assess the same (or similar) constructs. To conduct convergent validity analysis, our *a priori* hypothesis was that the PSEQ would have a significant negative correlation with fear-avoidance behaviors related to pain, assessed through the Tampa Scale of Kinesiophobia (TSK) (25), as shown in similar studies (12, 15, 17). This outcome has been previously associated with pain-self efficacy, and has been also recommended as a core pain-related assessment in pain clinical trials (33). Pearson’s correlation coefficient was used to establish correlation levels between these tools. As reported in similar studies that aimed to validate a translated version of the PSEQ (14, 18), absolute values above 0.3 are sufficient to support the tool’s validity.

#### 2.4.3. Test–retest reliability

Stability over time is assessed by conducting a test–retest reliability analysis, by assessing the same outcome twice in the same group of people and establishing the level of correlation between responses. To assess test–retest reliability, the Intraclass Correlation Coefficient (ICC) was used (model alpha, 2-way random effects model). ICC establishes a coefficient that ranges from 0 to 1, being 0 no correlation and 1 the highest correlation possible. Scores ranging from 0 to 0.4 are considered to have low correlation, from 0.4 to 0.6 moderate correlation, 0.6 to 0.8 average correlation and scores above 0.8 show excellent correlation (34). A subset of 40 participants was asked to complete again the PSEQ within a week after completing it for the first time. Additionally, a Bland–Altman graph was also created to plot the mean differences of the measurements with their limits of average difference corresponding limits ± the standard deviation’s difference (35).

Absolute reliability measures were also calculated through the standard error of measurement (SE) and the minimal detectable change (MDC). The MDC represents the smallest change in scores that can be considered beyond measurement error and is required to confidently conclude that a meaningful change has occurred in an individual’s pain self-efficacy. To calculate the SE, the following formula was used: SD × √(1-R), where SD is the Standard Deviation and R is the reliability coefficient of the instrument (36). To calculate the MDC, the following formula was used: 1.96 × √2× SE.

#### 2.4.4. Internal consistency

Internal consistency is the degree of relatedness between the items of an assessment tool (12). To assess the internal consistency of the PSEQ, Cronbach’s *α* was calculated. Cronbach’s *α* values range between 0 and 1, and an *α* value >0.9 was considered excellent, > 0.8 was considered good, and > 0.7 was considered acceptable (37). Corrected-total item correlation was also assessed to establish association levels between the items and the total score of the PSEQ.

## 3. Results

107 participants completed the questionnaire. Researchers in charge of the translation reached an agreement during the translation and adaptation process of the PSEQ into Spanish (PSEQ-Sp) and in a Spanish context. None of the items of the original were removed during the translation process.

### 3.1. Descriptive statistics

Demographic and clinical data of the study sample is shown in Table 1. Data was normally distributed. 112 participants met inclusion criteria and were enrolled in the study. However, 5 of them failed to complete all of the outcome measures and were excluded from the final analyses, leaving a total final sample of 107 participants. The sample included 48 males and 59 females, and the mean age was 49.4 years (SD 12.9). The vast majority of participants had a high educational level (97% had at least a High School degree), and were currently employed (91%).

**Table 1:** Baseline demographic characteristics from the sample.

Outcome	*n* (%) or mean (SD)
Gender (%)	
Male	48 (45%)
Female	59 (55%)
Age (years)	49.4 (12.9)
Involved side (%)	
Dominant	63 (59%)
Non-dominant	44 (41%)
Educational level (%)	
Uneducated	0 (0%)
Primary	3 (3%)
High School	41 (38%)
College	63 (59%)
Work status (%)	
Part-time	39 (36%)
Full-time	59 (55%)
Unemployed	7 (7%)
Retired	2 (2%)

The PSEQ-Sp mean score was 45.2 points (SD 11.4), and the TSK mean score was 46.6 points (SD 8.2). The PSEQ ranges from 0 to 60, and the TSK ranges from 17 to 68 points. None of the participants reported the lowest possible score 0, and only 3 (3%) of them reported the maximum score possible 60, so no significant floor and ceiling effects were found.

### 3.2. Validity

Results for the EFA are presented in Table 2. The value for the KMO assessing sampling adequacy was 0.90 and the score for Bartlett’s test was *X*^2^ = 1,866.08 (*p* < 0.001), suggesting that sampling was adequate, and data was appropriate for factor analysis. The exploratory factor analysis yielded for the PSEQ-Sp a one-factor solution, which accounted for 66% of the variance, as every item’s factor loading was >0.5. Additionally, every participant completed the PSEQ-Sp and the TSK measures, and the convergent validity analysis found a significant moderate negative correlation between the PSEQ-Sp and the TSK (Pearson’s correlation index *r* = −0.48, *p* < 0.001).

**Table 2:** Exploratory factor analysis results of the Spanish version of the PSEQ.

Description	Mean (SD)	Factor loading
Item 1. Puedo disfrutar de las cosas, a pesar del dolor	4.91 (1.31)	0.780
Item 2. Puedo realizar la mayoría de las tareas del hogar (recoger, lavar los platos…) a pesar del dolor	4.85 (1.80)	0.774
Item 3. Puedo socializar con amigos y familia tanto como solía hacer, a pesar del dolor	5.39 (1.20)	0.825
Item 4. Puedo gestionar mi dolor en la mayoría de las situaciones	4.40 (1.39)	0.811
Item 5. Puedo realizar alguna forma de trabajo a pesar del dolor (incluye trabajo doméstico, remunerado y no remunerado)	3.81 (2.82)	0.702
Item 6. Todavía puedo hacer muchas cosas que disfruto hacer, como hobbies o actividades de ocio, a pesar del dolor	4.53 (2.13)	0.882
Item 7. Puedo gestionar mi dolor sin medicación	4.95 (1.10)	0.644
Item 8. Todavía puedo alcanzar la mayoría de mis objetivos en la vida a pesar del dolor	4.84 (2.10)	0.776
Item 9. Puedo tener un estilo de vida normal a pesar del dolor	4.83 (2.43)	0.846
Item 10. Puedo volverme más activo gradualmente a pesar del dolor	4.11 (2.72)	0.758

### 3.3. Test–retest reliability

**Figure 1:**
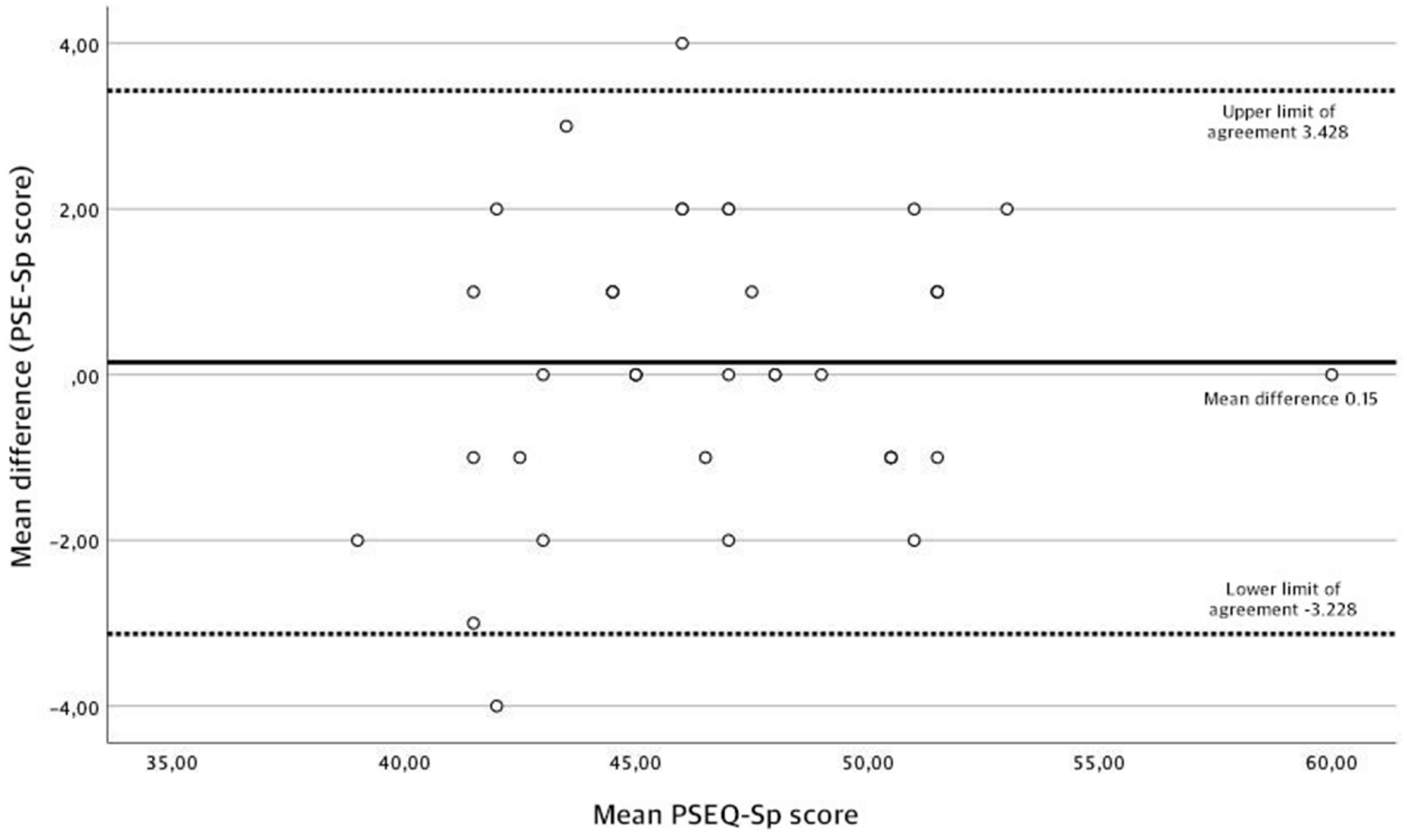
Results for the Bland-Altman plot graph.

Results for the test–retest reliability analysis are shown in Table 3. Every participant invited to respond to the PSEQ-Sp for a second time completed the questionnaire. The overall ICC for the PSEQ was 0.90 (95% CI 0.88–0.93), showing excellent correlation levels. ICC’s for each individual item ranged from 0.77 to 0.86. The SE was 1.23 and the MDC was 3.05 points, respectively. Additionally, most of the pair differences are between the agreement limits, as shown in the Bland–Altman plot graph (Figure 1). This implies that test–retest measure of the PSEQ-Sp have a high concordance.

**Table 3:** Descriptive statistics, internal consistency values, and intraclass correlations of the items in the PSEQ.

Description	Mean (SD)	ICC	95% CI	Cronbach’s *α* (if item deleted)	SE	MDC
Item 1. Puedo disfrutar de las cosas, a pesar del dolor	4.91 (1.31)	0.83	0.70–0.89	0.91		
Item 2. Puedo realizar la mayoría de las tareas del hogar (recoger, lavar los platos...) a pesar del dolor	4.85 (1.80)	0.79	0.63–0.88	0.92		
Item 3. Puedo socializar con amigos y familia tanto como solía hacer, a pesar del dolor	5.39 (1.20)	0.79	0.63–0.87	0.92		
Item 4. Puedo gestionar mi dolor en la mayoría de las situaciones	4.40 (1.39)	0.77	0.70–0.82	0.92		
Item 5. Puedo realizar alguna forma de trabajo a pesar del dolor (incluye trabajo doméstico, remunerado y no remunerado)	3.81 (2.82)	0.85	0.73–0.93	0.93		
Item 6. Todavía puedo hacer muchas cosas que disfruto hacer, como hobbies o actividades de ocio, a pesar del dolor	4.53 (2.13)	0.86	0.78–0.94	0.90		
Item 7. Puedo gestionar mi dolor sin medicación	4.95 (1.10)	0.80	0.70–0.88	0.93		
Item 8. Todavía puedo alcanzar la mayoría de mis objetivos en la vida a pesar del dolor	4.84 (2.10)	0.82	0.73–0.90	0.90		
Item 9. Puedo tener un estilo de vida normal a pesar del dolor	4.83 (2.43)	0.81	0.71–0.86	0.91		
Item 10. Puedo volverme más activo gradualmente a pesar del dolor	4.11 (2.72)	0.78	0.68–0.80	0.91		
Overall	45.2 (11.4)	0.90	0.88–0.93	0.92	1.23	3.05

### 3.4. Internal consistency

Results for the internal consistency analysis are shown in Table 3. The PSEQ-Sp showed overall excellent internal consistency, with a Cronbach’s α value of 0.92. Moreover, the Corrected-item total correlation values for Cronbach’s *α* if an item was deleted were also excellent, ranging from 0.90 to 0.93. These findings indicate that there is a strong association between the items and the total score.

## 4. Discussion

The aim of the present study was to translate and culturally adapt the Spanish version of the PSEQ, and examine its psychometric properties, being this the first study to perform such translation and analysis. Our results show that PSEQ-Sp has excellent test–retest reliability and excellent internal consistency. Also, our hypothesis stating that the PSEQ-Sp and the TSK were associated was confirmed, supporting the validity of the PSEQ-Sp.

The PSEQ is a relatively short and feasible questionnaire that can easily be administered during a regular assessment, and can provide valuable information to understand the patient’s clinical presentation from a biopsychosocial perspective. Authors have discussed its form and how can it be improved. For instance, respondents in the study conducted by Chala et al. (7) suggested to include every possible number in the scale, instead of including numbers only at every limit. However, authors in this study decided that such modification would have a significant influence in the performance of the scale, and opted not to conduct such modification.

The exploratory factor analysis yielded a one-factor solution, coinciding with previous studies (1, 11–19, 22, 23). Contrary to this, several studies did not report results for factorial analysis (20, 21, 24) and one study (7) showed a two-factor solution instead, alluding there may have been underlying factors in the cultural setting they assessed their translated version in Ethiopia. Also, the construct convergent validity of the PSEQ-Sp was assessed by performing a correlation analysis with the TSK. The moderate negative correlation found in our study between the PSEQ-Sp and the TSK is consistent with other studies that conducted the same analysis. Chiarotto et al. (17), Almutairi et al. (12), and Yang et al. (15) found moderate negative correlations of *r* = −0.48, −0.41, −0.45, respectively. All of these studies conducted their validity analysis in patients that suffered low back pain, unlike our study, but we can consider this positively, as the correlation between these tools seems to be consistent despite the clinical condition. However, our validity analysis could have been further developed by correlating the PSEQ with other tools that similar studies used, such as the Short-Form 36 (SF-36) assessing health-related quality of life (7, 12, 14, 18, 19, 23), or other indices of validity, such as discriminant (13), or factorial (7, 18) validity. Future studies could consider these analyses.

Both the Bland–Altman plot and ICC values showed excellent reliability levels for the PSEQ-Sp. Other studies have also used the ICC to establish correlation values for the PSEQ. The Arabic and Chinese-Hong Kong versions (12, 14) found average ICC correlation values of 0.79 and 0.75, respectively, and the Amharic, Canadian-French, Danish, Farsi, Italian, Japanese, and Marathi versions (1, 7, 16, 20) found high ICC correlation values that ranged from 0.80 to 0.96. However, we have to consider the possible incurring in a recall bias, as the high test–retest reliability might’ve been influenced by the interval between assessments.

The level of internal consistency of the PSEQ-Sp was excellent. Other language translations of the PSEQ have found excellent internal consistency levels too. The Amharic (7), Arabic (12), and Portuguese-Brazilian (22) versions had internal consistencies of *α* = 0.90, the Canadian-French (1) version of α = 0.91, the original English (11), Catalan (13), and Farsi (18) versions of *α* = 0.92, the Chinese-Hong Kong (14), and Marathi (20) versions of *α* = 0.93, the Italian (17), Japanese (19), and Mongolian (21) versions of *α* = 0.94, and the Chinese-Mainland (15) version of *α* = 0.95. The Portuguese-European (23) and Danish (16) versions had good levels of internal consistency, both *α* = 0.88. Only the Yoruba (24) version had inferior, but still acceptable levels of consistency of *α* = 0.79. Therefore, this tendency appears to be a psychometric property of the PSEQ across different languages and cultural contexts.

Limitations were also present in our study. First, results from this study are limited to patients suffering rotator cuff injuries, so conclusions should be interpreted cautiously. Also, our sample was of convenience, it was not randomly selected from the general population, meaning the generalizability cannot be assumed for all patients suffering rotator cuff injuries. Finally, we translated and cross-culturally adapted the PSEQ in Spain, and even though the version is easily understood by any Spanish speaker, cross-cultural adaptations to other Spanish-speaking countries, as the ones in South America, could report different results. Additional limitations to be considered include response biases, as our assessment was self-administered, and the potential recall bias on the test–retest assessment.

However, our study also presents strong points. This is the first translation of the PSEQ in Spanish, the world’s second most spoken native language, and the official language in 20 countries. Therefore, the development of the PSEQ-Sp could imply an important addition for so many clinicians and researchers. Future research could conduct cross-cultural adaptations of the PSEQ-Sp in different Spanish-speaking countries to explore potential variations in psychometric properties and cultural influences. Cross-cultural adaptations of the PSEQ-Sp in different Spanish-speaking countries to explore potential variations in psychometric properties and cultural influences.

## Data availability statement

The raw data supporting the conclusions of this article will be made available by the authors, without undue reservation.

## Ethics statement

The studies involving human participants were reviewed and approved by the Ethics Committee of University of Valencia (protocol code 2537824 and date of approval March 9, 2023). The patients/participants provided their written informed consent to participate in this study.

## Author contributions

BP-D and MB-D contributed to the conception and design of the study. SP-M and IE-P organized the database. AR-R performed the statistical analysis. BP-D wrote the first draft of the manuscript. All authors contributed to manuscript revision, read, and approved the submitted version.

## Conflict of interest

The authors declare that the research was conducted in the absence of any commercial or financial relationships that could be construed as a potential conflict of interest.

## Publisher’s note

All claims expressed in this article are solely those of the authors and do not necessarily represent those of their affiliated organizations, or those of the publisher, the editors and the reviewers. Any product that may be evaluated in this article, or claim that may be made by its manufacturer, is not guaranteed or endorsed by the publisher.
